# Impacts of a post-transport/pre-processing rest period on the growth performance, anthelmintic efficacy, and serum metabolite changes in cattle entering a feed yard

**DOI:** 10.1093/tas/txac085

**Published:** 2022-06-21

**Authors:** Payton L Dahmer, Charles A Zumbaugh, Macie E Reeb, Nicole B Stafford, Zachary T Buessing, Kenneth G Odde, James S Drouillard, A J Tarpoff, Cassandra K Jones

**Affiliations:** Kansas State University Department of Animal Sciences and Industry, Manhattan, KS 66506, USA; Kansas State University Department of Animal Sciences and Industry, Manhattan, KS 66506, USA; Kansas State University Department of Animal Sciences and Industry, Manhattan, KS 66506, USA; Kansas State University Department of Animal Sciences and Industry, Manhattan, KS 66506, USA; Kansas State University Department of Animal Sciences and Industry, Manhattan, KS 66506, USA; Kansas State University Department of Animal Sciences and Industry, Manhattan, KS 66506, USA; Kansas State University Department of Animal Sciences and Industry, Manhattan, KS 66506, USA; Kansas State University Department of Animal Sciences and Industry, Manhattan, KS 66506, USA; Kansas State University Department of Animal Sciences and Industry, Manhattan, KS 66506, USA

**Keywords:** anthelmintic efficacy, dry matter intake, growth performance, receiving calf

## Abstract

A total of 80 crossbred, high-risk heifers (initially 250 ± 4.2 kg BW), were transported from an Oklahoma City, Oklahoma sale barn to the Kansas State University Beef Cattle Research Center. Cattle were unloaded and randomly placed into one of four receiving pens and provided ad libitum hay and water. Each pen was randomly assigned to one of the four rest times before processing: (1) immediately upon arrival (0); (2) after a 6-h rest period (6); (3) after a 24-h rest period (24); and (4) after a 48-h rest period (48). After all cattle were processed, heifers were allotted into individual pens with ad libitum access to a receiving ration and water. Heifers were weighed individually on d 0, 7, 14, 21, 28, and 35 to calculate average daily gain (ADG). Feed added and refusals were measured daily to determine dry matter intake (DMI). A fecal egg count reduction test and analysis of blood serum metabolites were also conducted. All data were analyzed using the GLIMMIX procedure of SAS (v. 9.4, Cary, NC) with individual animal as the experimental unit. Processing time did not impact (*P* > 0.05) heifer BW or ADG. From d 0 to 35, DMI decreased linearly (*P* = 0.027) as rest time increased. The number of days for heifers to reach a DMI of 2.5% BW was linearly increased (*P* = 0.023) as rest time increased. There was no evidence of differences (*P* ≥ 0.703) among rest times for feed efficiency. While morbidity did not differ between treatments (*P* > 0.10), mortality increased linearly (*P* = 0.026) as the time of rest increased. A significant processing time × day interaction (*P* < 0.0001) was observed for the prevalence of fecal parasites, where the percentage of positive samples was significantly lower 14-d after anthelmintic treatment, regardless of the processing time. Serum IBR titer for heifers processed at either 0 or 6-h upon arrival was significantly higher (*P* < 0.01) on d 35 compared to d 0. Heifers processed after a 48-h rest period had significantly higher glucose values (*P* < 0.01) on d 0 compared to heifers processed at 0, 6, or 24-h. In summary, rest time prior to processing did not impact receiving calf growth performance. A 6-h rest period upon arrival appeared to be most beneficial to DMI. Anthelmintic treatment at processing reduced the parasitic load in heifers processed at all times. Vaccine titer did not increase after initial processing in heifers processed 24- or 48-h after arrival, indicating the seroconversion of IBR antibodies during the longer rest period.

## INTRODUCTION

Beef cattle are exposed to stress at multiple points throughout their life. While producers try to limit these instances, some, like transportation, are unavoidable. Transportation of cattle in the United States occurs in many facets such as movement through livestock auctions, to feedlots, and eventually to processing facilities. This means cattle can be transported once, up to five or more times in their lifetime (Schwartzkopf-Genswein and Grandin, 2014). The stress induced from transport can predispose calves to dehydration, reduced feed intake, inhibition of immune function, and increased susceptibility to bovine respiratory disease (BRD) (Van Engen et al., 2018). This disease, caused by both viral and bacterial agents, is responsible for substantial economic loss to the beef industry, totaling an estimated $1 billion, annually (NAHMS, 2013). Many methods have been adopted to decrease the severity of transport stress in newly received cattle. Preconditioning cattle by ensuring adequate weaning time prior to transport, vaccinating, castrating, dehorning, and treating with anthelmintics has been proven extremely effective (Duff and Galyean, 2007). In many cases, cattle are sourced from various locations and previous nutrient and health status is unknown. Therefore, management of cattle upon receiving also plays an integral role in their health and performance after arrival. Appropriately vaccinating, deworming, and treating with antibiotics is part of a successful receiving protocol. Likewise, providing newly received cattle with a nutrient-dense diet can combat their reduced feed intake (Loerch and Fluharty, 1999). Additionally, rest time during long transport of cattle has been studied, but data are variable regarding its benefits to animal stress levels and performance upon receiving (Cooke et al., 2013; Marti et al., 2017; Melendez et al., 2021). Delaying processing upon arrival to a feedlot is also an area of interest to counteract the stress associated with transport. Once received to a feedlot, cattle are typically placed into a receiving pen and allowed to rest, which is then followed by processing and placement into feedlot pens (Thomson et al., 2015). However, few studies have evaluated different rest times under controlled conditions. This lack of recent data prompted the current study, where we hypothesized that allowing calves rest time upon arrival would improve calf health and feedlot performance. Thus, our objectives were to evaluate the impact a post-transport rest period had on calf growth performance, mortality, and morbidity. This study also aimed to determine if a rest period affected calf response to anthelmintics and blood serum metabolites.

## MATERIALS AND METHODS

All experimental procedures adhered to the guidelines for the ethical and humane use of animals for research according to the Guide for the Care and Use of Agricultural Animals in Research and Teaching (FASS, 2010) and were approved by the Institutional Animal Care and Use Committee at Kansas State University (IACUC #4279).

### Animals and Experimental Design

A total of 80 crossbred heifers (initially 250 ± 4.2 kg BW) were transported approximately 482 km from an Oklahoma City, Oklahoma sale barn to the Kansas State University Beef Cattle Research Center (Manhattan, Kansas) via semi-truck, with a total transit time of approximately 6 h. Heifers were considered high-risk and originated from a geographic area high in parasites. Upon arrival, heifers were unloaded and as they came off the trailer were placed into one of four pens with free-choice water and alfalfa hay in a completely randomized design. Each pen of heifers (*n* = 20) was then randomly assigned to one of four treatments of varying rest times before processing: (1) immediately upon arrival (0); (2) after a 6-h rest period (6); (3) after a 24-h rest period (24); and (4) after a 48-h period (48). Processing was considered d 0 for the trial. At processing, all heifers were tagged, weighed, and subcutaneously injected with 1.0 mL/50 kg BW moxidectin (Cydectin, Bayer Animal Health, Shawnee Mission, KS) and orally dosed with 1.0 mL/50 kg BW oxfendazole (Synanthic, Boehringer Ingelheim Animal Health, St. Joseph, MO). Heifers were also subcutaneously injected with 1.1 mL/45 kg BW tulathromycin (Draxxin, Zoetis Animal Health, Parsippany, NJ), 2 mL of a recombinant *Mannheimia haemolytica* leukotoxoid vaccine (Nuplura PH, Elanco Animal Health, Greenfield, IN), and 2 mL of a modified-live virus vaccine (Titanium 5, Elanco Animal Health, Greenfield, IN) containing infectious bovine rhinotracheitis (IBR), bovine viral diarrhea (types 1 and 2), bovine respiratory syncytial virus, and parainfluenza 3. Finally, heifers were implanted with 140 mg of trenbolone acetate and 14 mg of estradiol (Revalor-H, Merck Animal Health, Whitehouse Station, NJ). After processing, cattle were returned to their receiving pen until all cattle had been processed at 48-h after arrival to the facility. Heifers were then placed into individual pens across two separate barns, with each pen containing an automatic waterer and feed bunk to provide ad libitum access to feed and water. Heifers were fed a standard receiving ration twice daily with feed refusals recorded. The diet was supplied as a total mixed ration (TMR), that met or exceeded all NASEM (2016) requirements. Diets consisted of 40% dry rolled corn, 30% ground alfalfa hay, 26% corn silage, and 4% receiving supplement (DM basis; Table 1). All animals were monitored daily for any health abnormalities. Any treatments were determined by staff in accordance with the facility’s standard operating procedures and all moralities had necropsies conducted at the Kansas State University Veterinary Diagnostic Laboratory (Manhattan, KS). All animals treated or removed from the trial were recorded, with morbidity analysis including first pull, second pull, third pull, and chronic.

**Table 1.** Ingredient composition and nutrient analysis of total mixed ration (TMR) fed to heifers from d 0 to d 35

Ingredient, % DM	TMR
Corn silage	26.0
Alfalfa hay, ground	30.0
Dry rolled corn	40.0
Receiving supplement^1^	4.0
Nutrient analysis, % DM	
Ether extract, %	3.13
Crude protein, %	12.50
Calcium, %	0.65
Phosphorous, %	0.29
Neutral detergent fiber, %	27.91
Acid detergent fiber, %	19.78

Receiving supplement was formulated with: ground corn (42.8%); soybean meal, dehulled (34.0%); urea, 46% N (9.3%); limestone (6.7%); salt (5.7%); Rumensin-90 (0.38%); and trace mineral premix (0.88%). Trace mineral premix provided: copper (250 mg/kg); manganese (499 mg/kg); selenium (2.46 mg/kg); zinc (749 mg/kg); vitamin A 0.17% (30,000 IU).

### Data Collection

Heifers were weighed individually on d 0, 7, 14, 21, 28, and 35 to calculate average daily gain (ADG). Feed was individually weighed and delivered to each heifer twice daily, with refusals collected and weighed daily to determine dry matter intake (DMI). On d 0 (processing) and d 35, blood samples were collected via the coccygeal vein from each heifer using sterile 15-mL vacutainer tubes (Vacutainer, Becton Dickinson, Franklin Lakes, NJ). Blood samples were immediately placed on ice and transported to the Kansas State University Veterinary Diagnostic Laboratory (Manhattan, KS) where serum was separated by centrifugation at 1,000 × *g* for 30 min and then analyzed for infectious bovine rhinotracheitis (IBR) titer via serum neutralization antibody test. Additionally, samples were analyzed for biochemical parameters via spectrophotometry using the Cobas c501 (Roche Diagnostics, Indianapolis, IN). To evaluate the efficacy of anthelmintic treatment, fresh fecal samples were collected via rectal grab on d 0 (processing) and d 14, placed on ice, and immediately transported to the Kansas State University Veterinary Diagnostic Laboratory for analysis of fecal parasites. First, semiquantitative analysis was conducted as described by Garcia et al. (2017), to determine the density of organisms in samples with a positive result. Using a microscope, each sample was given a density score according to the following: (1) rare/occasional (2–5 organisms per entire 22 × 22-mm coverslip area); (2) scanty/light/few (2 or fewer eggs or larvae/5 to 10 fields); (3) moderate (3–9 eggs or larvae/field); (4) numerous/heavy/many (10+ eggs or larvae/field). Then, a fecal egg count reduction test (FECRT) was conducted according to Gasbarre et al. (2009) using a modified Wisconsin Sugar Floatation Technique in order to determine the number of eggs per gram of feces. A subsample of 10 heifers/treatment was collected at processing and snap-frozen in liquid nitrogen for subsequent analysis.

### Statistical Analysis

All data were analyzed as a completely randomized design using the GLIMMIX procedure of SAS (v. 9.4, Cary, NC) with individual animal as the experimental unit. The statistical model included the random effects of ‘barn’ and ‘location within barn’. All comparisons included Tukey–Kramer multiple comparison adjustments. For growth performance, morbidity, and mortality data, pre-planned polynomial contrasts were conducted to evaluate linear and quadratic trends. For fecal parasite data and blood metabolite data, the model included the main effects of treatment and sampling day, as well as their interaction. Results were considered significant if *P* < 0.05 and a tendency if 0.05 < *P* < 0.10.

## RESULTS AND DISCUSSION

### Growth Performance, Mortality, and Morbidity

Growth performance, mortality, and morbidity data are presented in Table 2. Processing time did not impact (*P* ≥ 0.624) heifer BW or ADG for the duration of the experiment. From d 0 to d 14, there was a linear inverse relationship between DMI and time of rest (*P* = 0.012), where DMI decreased as the time of rest before processing increased. Likewise, for the overall experiment (d 0 to d 35), DMI decreased linearly (*P* = 0.027) as the rest time increased. Heifer DMI as a % of BW from d 0 to 14 decreased linearly (*P* = 0.020) as time of rest increased; however, this impact was only marginally significant (*P* ≥ 0.061) for the remainder of the trial. The number of days for heifers to reach a DMI of 2.5% BW was linearly increased (*P* = 0.023) as time of rest increased, with heifers processed at 0, 6, 24, or 48 h requiring 18, 15, 18, and 20 d to reach this parameter, respectively. The main effect of rest time significantly impacted (*P* = 0.038) the percentage of heifers that reached a targeted DMI of 2.5% BW by d 14 of the experiment, where 25.0%, 60.0%, 52.6%, and 23.5% of cattle reached this parameter after 0, 6, 24, and 48 h of rest prior to processing, respectively. Feed efficiency did not differ (*P* ≥ 0.70) between rest times. While morbidity did not differ between treatments (*P* > 0.10), mortality increased linearly (*P* = 0.026) as the time of rest increased. This increase was due to the loss of two experimental animals in the 48-h treatment on d 2 of the study.

**Table 2.** Impact of time of processing on feedlot heifer growth performance, mortality, and morbidity^1^

Item;	Processing time after arrival, h^2^				SEM	P =		
	0	6	24	48		Treatment	Linear	Quadratic
Weight, kg								
d 0	250	252	246	252	5.9	0.858	0.980	0.473
d 14	269	270	266	271	6.2	0.949	0.896	0.654
d 35	301	306	300	303	6.8	0.902	0.992	0.835
ADG, kg/d								
d 0–14	1.3	1.3	1.5	1.3	0.15	0.879	0.750	0.493
d 14–35	1.5	1.7	1.6	1.5	0.15	0.624	0.693	0.509
d 0–35	1.5	1.5	1.5	1.5	0.08	0.678	0.945	0.311
DMI, kg/d								
d 0–14	5.2^ab^	5.4^a^	5.1^ab^	4.9^b^	0.14	0.031	0.012	0.635
d 14–35	9.0	9.4	8.7	8.5	0.31	0.150	0.072	0.937
d 0–35	7.4	7.8	7.2	7.0	0.21	0.057	0.027	0.956
DMI, % of BW								
d 0–14	2.11	2.16	2.09	1.93	0.068	0.091	0.020	0.344
d 14–35	3.37	3.50	3.29	3.15	0.129	0.239	0.075	0.782
d 0–35	2.98	3.10	2.97	2.80	0.098	0.183	0.061	0.426
G:F								
d 0–14	0.25	0.24	0.29	0.26	0.030	0.645	0.507	0.368
d 14–35	0.17	0.18	0.18	0.18	0.015	0.891	0.626	0.936
d 0–35	0.20	0.20	0.21	0.21	0.010	0.703	0.375	0.471
Days to 2.5% BW DMI	18^ab^	15^b^	18^ab^	20^a^	1.3	0.030	0.023	0.393
Prevalence, %								
Mortality	0.0	0.0	0.0	10.5	3.57	0.096	0.026	0.236
Morbidity	0.0	0.0	5.3	0.0	2.60	0.382	0.806	0.113
Cattle to 2.5% BW by d 14	25.0	60.0	52.6	23.5	11.56	0.038	0.354	0.025

Means within a row that do not share a common superscript differ *P* < 0.05.

A total of 80 mixed-breed, high-risk heifers were used in a 35-d experiment with 1 heifer per pen and 20 replicates per treatment.

Cattle were processed at either 0, 6, 24, or 48 h after their arrival to the research facility.

Our data suggest that delaying processing time upon arrival does not impact growth performance of newly received feedlot cattle. However, processing cattle at 6-h upon arrival appeared to be the most beneficial to improve DMI. Heifers processed at 6-h had increased DMI for the duration of the experiment and took the fewest days to reach a targeted DMI of 2.5% BW. It is known that newly received cattle at a feed yard have reduced DMI (Loerch et al., 1999; Colombo et al., 2021). Calves that are healthy and unstressed can consume up to 3% of their BW, while high-risk, highly stressed cattle tend to consume 1.5% or less during the initial 2 weeks after receiving (Reinhardt and Thomson, 2015). Heifers in the current study were considered high-risk, but all cattle had a DMI well-beyond 1.5% BW two weeks into the experiment. By d 14, we saw a linear decrease in DMI as calves were processed beyond 6-h. Additionally, cattle were not allotted to their experimental pens until all calves had been processed at 48-h post-arrival. Each receiving pen provided free-choice hay and water, but no concentrate was provided until all cattle had been processed at 48-h. Much work has been done assessing how this time frame impacts the digestion and rumen function of cattle and their ability to adapt to the receiving diet (Duff and Gaylean, 2007; Gilbery et al., 2007; Smock et al., 2020). Limited ruminal fermentative capacity (RFC) is a potential factor responsible for limited feed intake in cattle deprived of feed due to transport. Several researchers have reported reduced ability of rumen microbes to ferment substrate after 48-h without feed (Baldwin, 1967; Cole and Hutcheson, 1981). Since all calves were feed-deprived for an equal amount of time, the increased DMI in calves processed at 6-h indicates their ability to adapt to the receiving diet sooner compared to their contemporaries in this study; however, we do not have a clear explanation for why this occurred. Unfortunately, other parameters like RFC or digestibility were not measured to assess a potential mechanism of action behind the increase in DMI in heifers processed at 6-h. An important limitation of the current work was how cattle were fed. Unlike a traditional feedlot setting, heifers were penned and fed individually, which is not completely indicative of normal industry practice.

### Anthelmintic Efficacy

Fecal parasitic data are presented in Table 3. The percentage of positive samples for overall fecal parasite prevalence was significantly lower (*P* < 0.0001) 14-d after anthelmintic treatment when compared to the count on d 0. The processing time × day interaction was significant (*P* ≤ 0.04) for the semiquantitative density of Srongyle, Eimeria, Trichuris, and Strongyloides organisms, where their density was significantly lower 14-d after anthelmintic treatment. Finally, only Strongyle and Eimeria eggs were identified using the FECRT, and the processing time × day interaction was again significant (*P* ≤ 0.01), where the number of eggs per gram of feces was lower on d 14 compared to d 0.

**Table 3.** Impact of processing time after arrival on feedlot heifer fecal parasites at d 0 and 14 d after anthelmintic administration^1^

Item	Processing time after arrival, h^2^				SEM	Day, *P*
	0	6	24	48		
Prevalence, %					8.69	<0.0001
d 0	94.7^a^	90.0^a^	100.0^a^	93.3^a^		
d 14	21.1^b^	20.0^b^	11.1^b^	40.0^b^		
Parasitic load, semiquantitative density^3^						
Strongyle					0.25	<0.0001
d 0	2.7^a^	3.0^a^	2.8^a^	2.6^a^		
d 14	0.2^b^	0.4^b^	0.1^b^	0.2^b^		
Eimeria					0.19	<0.0001
d 0	2.5^a^	2.3^a^	2.9^a^	2.0^ab^		
d 14	0.4^b^	0.4^b^	0.3^bc^	0.2^c^		
Trichuris					0.09	<0.0001
d 0	2.4^a^	2.5^a^	2.6^a^	2.4^a^		
d 14	0.3^b^	0.4^b^	0.3^b^	0.2^b^		
Strongyloides					0.019	0.043
d 0	0.06^a^	0.07^a^	0.06^a^	0.05^a^		
d 14	0.00^b^	0.00^b^	0.00^b^	0.00^b^		
Moniezia					0.031	0.297
d 0	0.04	0.03	0.01	0.08		
d 14	0.01	0.02	0.00	0.00		
Giardia					0.051	0.084
d 0	0.11	0.09	0.10	0.13		
d 14	0.00	0.01	0.00	0.05		
Parasitic load, eggs/g of feces^4^						
Strongyle					128.0	0.0009
d 0	263^a^	261^a^	411^a^	325^a^		
d 14	1^b^	6^b^	0^b^	1^b^		
Eimeria					97.2	<0.0001
d 0	135^a^	129^a^	204^a^	152^a^		
d 14	4^b^	1^b^	15^b^	6^b^		

Means within response criteria that do not share a common superscript differ *P* < 0.05.

A total of 80 mixed-breed, high-risk heifers were used in a 35-d experiment with 1 heifer per pen and 20 replicates per treatment.

Cattle were processed at either 0, 6, 24, or 48 h after their arrival to the research facility.

Semiquantitative analysis was conducted as described by Garcia et al. (2017). Scores indicated: (1) rare/occasional: 2–5 organisms per entire 22 × 22-mm coverslip area; (2) scanty/light/few: 2 or fewer eggs or larvae/5–10 fields; (3) moderate: 3–9 eggs or larvae/field; (4) numerous/heavy/many: 10+ eggs or larvae/field.

On d 0 and d 14 a fecal egg count reduction test was conducted to determine the number of eggs per g of feces as an indication of anthelmintic efficacy.

We hypothesized that anthelmintic treatment at processing would reduce the presence of fecal parasites by d 14, regardless of when cattle were processed upon arrival. The World Association for Advancement of Veterinary Parisitology (WAAVP) has established guidelines for conducting FECRT, and suggests that anthelmintic efficacy be determined at a 90% reduction threshold (Coles et al., 1992). The presented data indicate that treatment with both moxidectin and oxfendazole was effective at reducing fecal parasites. Cattle across all treatments were at or above this threshold at initial sampling on d 0 (94.7%, 90.0%, 100.0%, and 93.3% prevalence for the 0, 6, 24, and 48-h processing times, respectively), but were significantly reduced and fell below the 90% threshold by sampling on d 14 (21.1%, 20.0%, 11.1%, and 40.0% prevalence for the 0, 6, 24, and 48-h processing times, respectively). These results were expected and coincide with other work studying anthelmintic efficacy (Utley et al., 1974; Ives et al., 2007; Fazzio et al., 2016). Gastrointestinal parasitism is a leading cause of reduced performance in newly received feedlot cattle, and prior environment plays a large role in this, as cattle that have been backgrounded on pasture have increased exposure to larvae which can prompt further infections (Griffin et al., 2018). The heifers in the current study originated from a geographic location high in parasites; therefore, the large parasite burden on d 0 was expected. Parasitic infections can lead to reduced intake, digestibility, and other physiological mechanisms which can thereby negatively impact animal health, performance, and economic efficiency (Perry et al., 1999). Because it is well established that anthelmintic treatment is often effective, producers should use tools like FECRT to determine their treatment protocols. Additionally, future work evaluating anthelmintic treatments at varying processing times and anthelmintic resistance should be done.

### Blood Serum Metabolites

Serum metabolite data are presented in Table 4. While a significant processing time × day interaction was observed for nearly all parameters (*P* < 0.05), only few differences were biologically significant. Serum IBR titer for heifers processed at either 0 or 6-h upon arrival was significantly higher (*P* < 0.01) on d 35 compared to d 0. This response was expected, as these cattle were vaccinated immediately or shortly after arrival. Interestingly, no difference in IBR titer was observed (*P* > 0.05) between d 0 and d 35 for heifers processed at either 24 or 48-h upon arrival, indicating that these cattle may have been exposed to virus during transport or the rest period, and had time to seroconvert antibodies to the virus before vaccination. Heifers processed after a 48-h rest period had significantly higher glucose values (*P* < 0.01) on d 0 compared to heifers processed at 0, 6, or 24-h; however, this parameter was standardized across processing treatments by d 35. Other researchers have found that transit stress can result in increased blood glucose (Galyean et al., 1981; Damtew et al., 2018), thus, the concentration found in heifers processed 48-h upon arrival might suggest this rest period prompted more stress on the animals. However, the current work did not look at more stress-specific hormones, such as cortisol, which could provide explanation to the observed glucose differences and stress levels. Additionally, heifers across all processing times had increased (*P* < 0.0001) sorbitol dehydrogenase (SDH) from d 0 to d 35. While increases in SDH are most commonly associated with hepatocellular injury, the observed levels were not outside of normal biological ranges.

**Table 4.** Impact of processing time after arrival on IBR titer and serum biochemical parameters^1^

	Processing time after arrival, h^2^				SEM	Treatment × day, *P*
	0	6	24	48		
Blood parameter						
IBR Titer, 1:X^3^					15.2	0.0006
d 0	8^b^	1^b^	54^ab^	54^ab^		
d 35	64^a^	70^a^	47^ab^	31^ab^		
Glucose, mg/dL					7.3	0.0002
d 0	82^bc^	76^bc^	68^c^	108^a^		
d 35	83^bc^	85^abc^	83^abc^	96^ab^		
Urea Nitrogen, mg/dL					0.9	<0.0001
d 0	12^b^	18^a^	16^a^	17^a^		
d 35	9^b^	10^b^	10^b^	9^b^		
Creatinine, mg/dL					0.10	0.0008
d 0	1.2^ab^	1.2^ab^	1.2^ab^	1.3^a^		
d 35	0.9^b^	0.9^b^	1.0^b^	1.1^ab^		
Total Protein, g/dL					0.15	<0.0001
d 0	7.4^a^	7.4^a^	7.3^ab^	7.3^ab^		
d 35	6.7^c^	6.7^c^	6.8^bc^	6.8^bc^		
Albumin, g/dL					0.07	0.563
d 0	3.3	3.2	3.3	3.4		
d 35	3.3	3.3	3.2	3.2		
Globulin, g/dL					0.15	<0.0001
d 0	4.1^a^	4.1^a^	4.0^ab^	3.9^abc^		
d 35	3.4^cd^	3.4^d^	3.6^bcd^	3.6^bcd^		
Total Ca, mg/dL					10.12	0.0002
d 0	9.2^bc^	9.1^c^	9.2^bc^	10.1^a^		
d 35	9.7^abc^	9.6^abc^	9.6^abc^	9.9^ab^		
P, mg/dL					0.39	<0.0001
d 0	8.5^b^	10.2^a^	8.8^ab^	7.9^b^		
d 35	7.7^bc^	8.0^bc^	7.9^bc^	7.0^c^		
Na, mmol/L					0.7	0.0005
d 0	145^a^	143^b^	143^b^	143^b^		
d 35	142^b^	142^b^	142^b^	143^ab^		
K, mmol/L					6.39	<0.0001
d 0	5.7^b^	5.5^b^	6.4^a^	5.9^ab^		
d 35	5.5^b^	5.2^b^	5.5^b^	5.7^b^		
Cl, mmol/L					0.8	<0.0001
d 0	104^a^	100^b^	96^c^	94^c^		
d 35	96^c^	97^c^	96^c^	97^c^		
Bicarbonate, mmol/L					1.1	0.0008
d 0	19^b^	22^ab^	22^ab^	18^b^		
d 35	22^ab^	23^a^	23^a^	22^ab^		
Anion Gap, mmol/L					1.2	<0.0001
d 0	29^bc^	27^c^	32^b^	37^a^		
d 35	30^bc^	29^bc^	30^bc^	30^bc^		
Na:K Ratio					0.7	<0.0001
d 0	26^a^	26^a^	23^b^	25^ab^		
d 35	26^a^	27^a^	26^a^	26^ab^		
Aspartate transaminase P5P, U/L					14.2	0.255
d 0	127	118	134	123		
d 35	140	105	111	100		
Alkaline phosphatase, U/L					17.5	<0.0001
d 0	112^c^	120^c^	142^bc^	119^c^		
d 35	208^a^	204^ab^	199^ab^	201^ab^		
Gamma glutamyl transferase, U/L					1.7	0.016
d 0	9^ab^	9^ab^	5^b^	7^ab^		
d 35	10^ab^	9^ab^	10^ab^	13^a^		
Sorbitol dehydrogenase, U/L					2.18	<0.0001
d 0	6.5^b^	10.2^b^	3.6^b^	4.5^b^		
d 35	20.9^a^	18.7^a^	18.2^a^	19.5^a^		
Creatine kinase, U/L					825	0.375
d 0	1,360	1,094	1,041	1,625		
d 35	2,889	694	1,631	605		
Total bilirubin, mg/dL					0.02	<0.0001
d 0	0.2^ab^	0.2^bc^	0.3^a^	0.2^bc^		
d 35	0.1^c^	0.1^c^	0.1^c^	0.1^c^		
Direct bilirubin, mg/dL					0.01	0.058
d 0	0.1	0.1	0.1	0.1		
d 35	0.1	0.1	0.1	0.1		

Means within the same row that do not share a common superscript differ, *P* < 0.05.

A total of 80 mixed-breed, high-risk heifers were used in a 35-d experiment with 1 heifer per pen and 20 replicates per treatment.

Cattle were processed at either 0, 6, 24, or 48 h after their arrival to the research facility.

Serum samples were analyzed for infectious bovine rhinotracheitis (IBR) titer via serum neutralization antibody test with the means displayed as the ratio of serum: dilutant where no antibodies remained detectable within the sample.

In summary, rest time prior to processing did not impact receiving calf growth performance. These data suggest that 6 h, or approximately 1 h of rest per hour of transport time, was the most beneficial to maximizing DMI during the first 14 d after arrival to the feedlot. Anthelmintic treatment at processing reduced the parasitic load in all heifers, regardless of their rest time upon arrival. Vaccine titer did not increase after initial processing in heifers processed 24- or 48-h after arrival, indicating the seroconversion of IBR antibodies during the longer rest period. Continued research with increased replication and more industry-standard experimental conditions should be conducted to further validate how rest time prior to processing can affect the health and growth performance of cattle entering a feed yard.
